# Crohn’s Disease is a Greater Risk Factor for Nonalcoholic Fatty Liver Disease Compared to Ulcerative Colitis: A Systematic Review

**DOI:** 10.7759/cureus.42995

**Published:** 2023-08-05

**Authors:** Athri Kodali, Chiugo Okoye, Dhadon Klein, Iman Mohamoud, Olawale O Olanisa, Panah Parab, Priti Chaudhary, Sonia Mukhtar, Ali Moradi, Pousette Hamid

**Affiliations:** 1 Internal Medicine, California Institute of Behavioral Neurosciences and Psychology, Fairfield, USA; 2 Medicine, Saint James School of Medicine, Park Ridge, USA; 3 Endocrinology and Diabetes, Queens Hospital Center, New York City, USA; 4 Internal Medicine, Lahore Medical and Dental College, Lahore, PAK; 5 Medicine, Semmelweis University, Budapest, HUN; 6 Neurology, California Institute of Behavioral Neurosciences and Psychology, Fairfield, USA

**Keywords:** nafld, nonalcoholic steatohepatitis, risk factors for nonalcoholic fatty liver disease, nonalcoholic fatty liver disease (nafld), (ibd) inflammatory bowel disease, ulcerative colitis (uc), crohn’s disease (cd)

## Abstract

Crohn's disease (CD) and ulcerative colitis (UC) are inflammatory bowel diseases that have been associated with nonalcoholic fatty liver disease (NAFLD). This systematic review aimed to examine whether Crohn's disease confers a greater risk for nonalcoholic fatty liver disease compared to ulcerative colitis. A comprehensive search of electronic databases from January 2000 to May 2023 was conducted to identify observational studies investigating the association between Crohn's disease or ulcerative colitis and nonalcoholic fatty liver disease. Preferred Reporting Items for Systematic Reviews and Meta-Analysis (PRISMA) 2020 checklist ensured transparent reporting, and the Newcastle-Ottawa Scale was used to assess study quality. Data synthesis revealed higher nonalcoholic fatty liver disease prevalence among Crohn's disease patients compared to ulcerative colitis patients across regions. Ten studies published between 2016 and 2022, encompassing a total of 4164 participants from three continents, were included in the review. The median proportion of Crohn's disease patients with nonalcoholic fatty liver disease was 37.22% (range: 10.95-53.80%), while it was 27.55% (range: 8.60-46.20%) for ulcerative colitis patients. Subgroup analysis by region confirmed CD's higher NAFLD risk. Median proportions for CD patients who developed NAFLD from North America, Europe, and Asia were 25.97% (range: 14.6-37.33%), 47.01% (range: 14.2-53.8%), and 20.78% (range: 10.95-30.6%), respectively, and the median proportion of persons with UC who developed NAFLD in studies from North America, Europe, and Asia were 17.28% (range: 8.6-25.96%), 37.70% (range: 25.64-46.20%), and 19.52% (range: 10.14-28.90%), respectively. Variations suggest differing mechanisms, disease features, and therapeutics. Transmural inflammation in Crohn's disease may increase metabolic abnormalities, including nonalcoholic fatty liver disease. Geographic differences in lifestyle, genetics, and environmental variables may also contribute. This review demonstrates that Crohn's disease patients face a higher nonalcoholic fatty liver disease risk than ulcerative colitis patients, emphasizing the need for early monitoring and prevention. Further studies are warranted to understand mechanisms and develop tailored management approaches.

## Introduction and background

Crohn's disease (CD) and ulcerative colitis (UC) are two major subtypes of inflammatory bowel disease (IBD) that affect millions of individuals worldwide [1]. These chronic conditions not only pose significant challenges to patients' gastrointestinal health but also have been associated with various extra-intestinal manifestations. One such manifestation is the increased risk of developing nonalcoholic fatty liver disease (NAFLD), a prevalent chronic liver condition characterized by fat buildup in the liver without considerable alcohol consumption [2]. NAFLD is considered a global health concern due to its rising prevalence and potential for progression to more severe liver-related complications, such as nonalcoholic steatohepatitis (NASH), cirrhosis, and hepatocellular carcinoma [3].

Despite the emerging evidence on the topic, there is still a lack of agreement regarding the magnitude of the association between CD or UC and NAFLD. Some studies suggest a higher prevalence of NAFLD in CD patients, while others indicate a similar or even higher prevalence in UC patients [2,4-12]. The underlying mechanisms for the development of NAFLD in IBD patients remain incompletely understood [6]. It is believed that the chronic systemic inflammation characteristic of CD and UC may contribute to the development and progression of liver steatosis [13]. Additionally, factors such as altered gut microbiota, genetic predisposition, dietary habits, and lifestyle factors may further modulate the risk of NAFLD in these patients [14].

Understanding the differences in the effects of CD and UC on NAFLD risk is critical for doctors, researchers, and policymakers. Early detection, risk stratification, and targeted therapies can all benefit from an accurate assessment of NAFLD prevalence in CD and UC patients. Furthermore, evaluating potential differences in NAFLD risk between CD and UC across geographic locations can provide valuable insights into the impact of genetic, environmental, and cultural factors on disease outcomes.

Therefore, a comprehensive and systematic review synthesizing the available evidence is warranted to provide a clearer understanding of whether CD or UC confers a greater risk for NAFLD. The purpose of this systematic review was to evaluate and analyze the available literature on the prevalence of NAFLD in CD and UC patients, compare the risk between the two conditions, explore potential geographic disparities, and discuss potential reasons leading to the observed variances. This study attempts to offer a comprehensive overview of existing knowledge and highlight gaps that require additional investigation by compiling and critically evaluating data from numerous publications.

This systematic review's findings will have significant consequences for clinical practice, patient management, and future research endeavors. The findings may alert healthcare practitioners to the importance of vigilant monitoring and management of NAFLD in CD and UC patients. Furthermore, identifying geographic inequalities in NAFLD risk can help to lead the development of region-specific strategies for early intervention, lifestyle changes, and targeted therapeutics. Finally, this systematic review will help to gain a better understanding of the link between CD, UC, and NAFLD, paving the way for better patient care and more informed decision-making in the field of gastroenterology and hepatology.

## Review

Methods

This systematic review follows the guidelines outlined in the Preferred Reporting Items for Systematic Reviews and Meta-Analyses (PRISMA) 2020 statement [15]. The purpose of this systematic review is to evaluate and analyze the available literature on the prevalence of nonalcoholic fatty liver disease in Crohn's disease and ulcerative colitis patients, compare the risk between the two conditions, explore potential geographic disparities, and discuss potential reasons leading to the observed variances. The population, exposure (intervention), control group, and outcome (PECO) description was used as an organizing framework for the study question to ensure a priori establishment of the study methodology [16].

Search Strategy

A thorough literature search was carried out utilizing the electronic databases MEDLINE/PubMed, PubMed Central, and Google Scholar, from January 2000 to May 2023, to identify all pertinent articles. Medical Subject Headings (MeSH) terms “inflammatory bowel disease,” “Crohn's disease,” “colitis, ulcerative,” “nonalcoholic fatty liver disease,” and “epidemiology” were used in different combinations to generate a comprehensive and up-to-date list of articles. In addition, thorough searches of reference lists and pertinent journals were conducted to verify that all relevant papers were included. Two independent reviewers conducted the search and selected the articles for final evaluation. Any differences were settled by mutual discussion and screening by a third reviewer using a modified Delphi system [17]. The references of the initially found papers were then manually checked to find additional research that might have been missed during the original search.

Study Selection

The articles from the search results were screened in a three-step process. Initially, search filters were used to narrow down the articles based on the criteria. The titles and abstracts were then evaluated for their relevance and eligibility. Following that, full-text papers from potentially eligible studies were evaluated for ultimate inclusion. Inclusion criteria encompassed studies that evaluated the prevalence of NAFLD in patients with CD and UC, provided sufficient data for analysis, and were published in English. Studies focusing on pediatric populations, case reports, reviews, and editorials were excluded.

Data Collection

Two reviewers worked independently to extract data using a standardized data extraction form and any discrepancy was resolved by a third reviewer using a modified Delphi system [17]. The following data were gathered from each included study: author(s), year of publication, study design, study duration, country, region, sample size, diagnostic method for NAFLD, and prevalence rates of NAFLD in CD and UC patients.

Quality Assessment

The Newcastle-Ottawa Scale (NOS) was employed to evaluate the quality of the included observational studies. The NOS evaluates three following domains: selection of study groups, comparability of groups, and ascertainment of exposure/outcome. Each study is awarded a maximum of nine stars based on the quality of these domains. Studies with scores of seven or more are considered high quality, scores of four to six are considered moderate quality, and scores below four are considered low quality [18]. Two reviewers did the quality assessment independently and any discrepancies were resolved through discussion.

Data Synthesis and Statistical Analysis

The extracted data were synthesized qualitatively to provide a descriptive overview of the prevalence of NAFLD in CD and UC patients across different studies and regions. To study potential variations in NAFLD risk, the findings were grouped by geographic area (North America, Europe, and Asia). A meta-analysis was not performed because of the heterogeneity of the included observational studies.

Results

Study Selection

**Figure 1:**
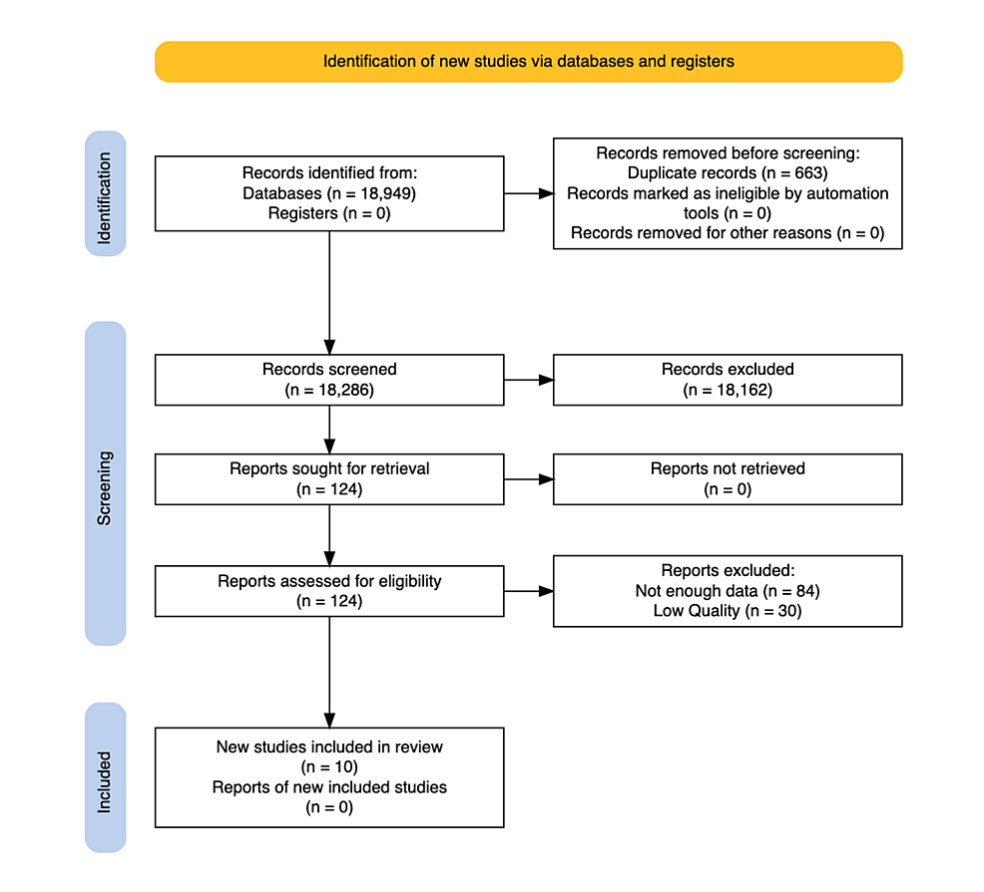
PRISMA statement diagram delineating the process by which articles were screened and ultimately included PRISMA: Preferred Reporting Items for Systematic Reviews and Meta-Analyses

The literature search identified 18,949 articles of which 18,286 were screened after removing duplicates. Of these, 18,162 were excluded based on the various inclusion and exclusion criteria. Only 124 were deemed eligible for further assessment. Of these, 84 studies did not have enough data and 30 studies were of low quality and were thus excluded. The remaining 10 studies met inclusion criteria and were deemed eligible for data analysis. The study selection process is summarized using a PRISMA flow diagram to provide a transparent representation of the study selection (Figure 1) [15].

Study Characteristics

**Figure 2:**
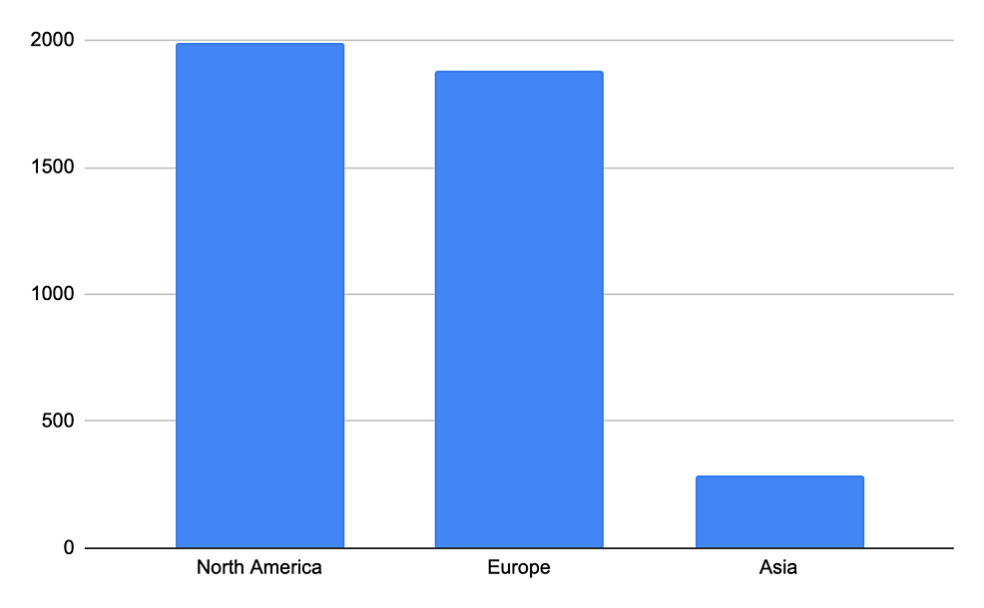
Region-wise distribution of number of study participants. The image is created by the authors of this study.

The included observational studies covered a wide range of geographic regions, including Europe, North America, and Asia. The study designs varied, with most studies being retrospective in nature. The sample sizes of the observational studies included ranged from 81 to 1672, with a median sample size of 165. Diagnostic criteria for CD, UC, and NAFLD varied across studies, with some studies relying on clinical criteria, while others utilized imaging or histological confirmation. Table 1 below summarizes the characteristics of the studies including the study country, study period, participant size, NAFLD prevalence in CD patients, and NAFLD prevalence in UC patients. Figure 2 below shows that more studies are needed in Asia [2,4-12].

**Table 1:** Baseline characteristics of the included studies. NAFLD: nonalcoholic fatty liver disease; CD: Crohn's disease; UC: ulcerative colitis

Study	Country	Region	Period	Number of participants	Percentage of CD patients with NAFLD	Percentage of UC patients with NAFLD
Sartini et al. [2]	Italy	Europe	March 2012 to March 2016	223	53.80%	46.20%
Bessissow et al. [4]	Canada	North America	2006 to 2013	321	37.33%	25.96%
Ritaccio et al. [5]	USA	North America	2007 to 2017	1672	14.60%	8.60%
Dias et al. [6]	Portugal	Europe	2010 to 2020	101	37.10%	25.64%
Hoffmann et al. [7]	Germany	Europe	June 1, 2014 to May 31, 2018	694	48.01%	44.44%
Fousekis et al. [8]	Greece	Europe	1977 to 2016	602	14.20%	26.20%
Kani et al. [9]	Turkey	Europe	November 2012 to April 2017	99	48.30%	38.50%
Yen et al. [10]	Taiwan	Asia	January 2019 to December 2019	81	30.60%	28.90%
Chicco et al. [11]	Italy	Europe	January 2018 to December 2018	165	46%	36.90%
Li et al. [12]	China	Asia	January 1, 2012 to May 1, 2016	206	10.95%	10.14%

Prevalence of Nonalcoholic Fatty Liver Disease in Crohn's Disease and Ulcerative Colitis Patients

Worldwide: The prevalence of NAFLD in CD and UC patients was assessed across the 10 studies published between 2016 and 2022. The study period for these studies was between 1977 and 2020. The median sample size was 165 (range: 81-1672). The median proportion of persons with CD who developed NAFLD was 37.22% (range: 10.95%-53.80%). The median proportion of persons with UC who developed NAFLD was 27.55% (range: 8.60-46.2%). Except for one study in Greece that followed patients from 1977 to 2016, in all other studies persons with CD had a higher risk of developing NAFLD than persons with UC. Notably, the prevalence rates varied across studies and geographic regions.

North America: Two North American studies published between 2016 and 2020 were included (one in the United States and one in Canada). The study period for these studies was between 2006 and 2017. The median sample size was 996.5 (range: 321-1672). The median proportion of persons with CD who developed NAFLD was 25.97% (range: 14.60-37.33%). The median proportion of persons with UC who developed NAFLD was 17.28% (range: 8.60-25.96%). In all the studies, persons with CD had a higher risk of developing NAFLD than persons with UC.

Europe: Six European studies published between 2018 and 2022 were included spanning the following countries in Europe: Germany, Greece, Portugal, Italy, and Turkey. The study period for these studies was between 1977 and 2020. The median sample size was 194 (range: 99-694). The median proportion of persons with CD who developed NAFLD was 47.01% (range: 14.20-53.8%). The median proportion of persons with UC who developed NAFLD was 37.70% (range: 25.64-46.20%). In five studies, persons with CD had a higher risk of developing NAFLD and in one study in Greece, persons with UC had a higher risk of developing NAFLD.

Asia: Two Asian studies published between 2017 and 2021 were included from China and Taiwan. The study period for these studies was between 2012 and 2019. The median sample size was 143.5 (range: 81-206). The median proportion of persons with CD who developed NAFLD was 20.78% (range: 10.95-30.6%). The median proportion of persons with UC who developed NAFLD was 19.52% (range: 10.14-28.90%).

These findings suggest a consistent pattern across various regions, with CD patients generally exhibiting a higher risk of developing NAFLD compared to UC patients. However, it is important to note the variations in NAFLD prevalence within each group and across different geographic regions.

The results of this systematic review provide valuable insights into the association between CD and UC with NAFLD. These findings validate the necessity for additional research to understand the underlying mechanisms and explore potential interventions to mitigate the increased risk of NAFLD in CD patients. In addition, healthcare professionals should consider the heightened risk of NAFLD in CD patients during clinical management and implement appropriate monitoring and preventive strategies to address this comorbidity.

Quality Assessment

The quality of the observational studies included was assessed using the Newcastle-Ottawa Scale (NOS). The scores ranged from 6 to 9, indicating high methodological quality. The studies demonstrated appropriate selection of study groups, comparability of groups, and adequate ascertainment of exposure/outcome.

*Synthesis of Findings* 

Given the heterogeneity among the included studies, a meta-analysis was not feasible. Instead, a qualitative synthesis of the findings was conducted, highlighting the variations in NAFLD prevalence between CD and UC patients across different studies and regions (Table 2) [2,4-12]. The results indicate that CD may confer a higher risk for NAFLD compared to UC, although the magnitude of this association varied across studies and geographic locations.

**Table 2:** Region-based variation in the prevalence of NAFLD in Crohn’s disease patients and ulcerative colitis patients. NAFLD: nonalcoholic fatty liver disease; CD: Crohn's disease; UC: ulcerative colitis

Region	Number of study participants (total, median, range)			NAFLD in CD patients (median, range)		NAFLD in UC patients (median, range)	
Worldwide	4164	165	81-1672	37.22%	53.80-10.95%	27.55%	8.60-46.2%
North America	1993	996.5	321-1672	25.97%	14.60-37.33%	17.28%	8.60-25.96%
Europe	1884	194	99-694	47.01%	14.20-53.8%	37.70%	25.64-46.20%
Asia	287	143.5	81-286	20.78%	10.95-30.6%	19.52%	10.14-28.90%

Discussion

The present systematic review examined the association between Crohn's disease (CD) and ulcerative colitis (UC) with nonalcoholic fatty liver disease (NAFLD) based on the 10 observational studies conducted worldwide between 2016 and 2022, encompassing a study period ranging from 1977 to 2020 [2,4-12]. The included studies had a median sample size of 165 individuals, with a range of 81-1672 participants. The results revealed important insights into the prevalence of NAFLD in CD and UC patients across different geographic regions and highlighted potential disparities in the impact of these inflammatory bowel diseases on NAFLD.

**Figure 3:**
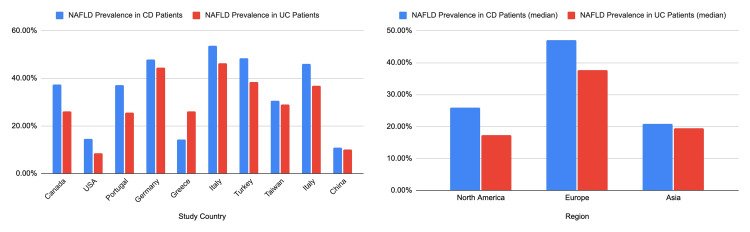
Comparison of NAFLD prevalence in CD patients versus UC patients by country and region. NAFLD: nonalcoholic fatty liver disease; CD: Crohn's disease; UC: ulcerative colitis The image is created by the authors of this study.

Based on this systematic review, the results indicate that CD may confer a higher risk for NAFLD compared to UC. The median proportions of CD patients developing NAFLD ranged from 10.95% to 53.80%, with a median of 37.22%. In contrast, the median proportions of UC patients developing NAFLD ranged from 8.60% to 46.20%, with a median of 27.55%. These results in Figure 3 indicate a consistently higher prevalence of NAFLD among CD patients across the included studies [2,4-12].

Geographic disparities were evident in the prevalence rates of NAFLD among CD and UC patients. In North America, CD patients demonstrated a median proportion of NAFLD at 25.97%, whereas UC patients showed a lower median proportion of 17.28%. Similar trends were observed in Europe, where CD patients had a median proportion of NAFLD at 47.01%, while UC patients had a slightly lower median proportion of 37.70%. In Asia, the prevalence of NAFLD in both CD and UC patients was relatively lower, with median proportions of 20.78% and 19.52%, respectively. These findings also suggest a comparable risk of NAFLD development between individuals with CD and UC in the Asian population.

It is important to consider the potential factors contributing to the observed disparities in the impact of CD and UC on NAFLD. One possible explanation is the differences in disease characteristics and inflammatory processes between CD and UC. The distinct pathophysiological mechanisms underlying CD and UC could contribute to variations in NAFLD development. CD is characterized by transmural inflammation and can affect any region of the gastrointestinal tract, including the small intestine, whereas UC is limited to the colon and involves superficial inflammation [19]. The variation in the anatomical distribution and severity of inflammation in CD may contribute to a higher risk of metabolic disturbances, including NAFLD. Furthermore, disparities in CD and UC management strategies, including treatments and surgical interventions, may play a role in the observed differences in NAFLD prevalence. The use of aggressive medical therapies, including immunosuppressive therapies, corticosteroids, and biological agents in CD management may have varying effects on metabolic health and liver function, potentially influencing the development of NAFLD [20,21]. Differences in surgical interventions, such as bowel resection or colectomy, between CD and UC patients may also impact NAFLD prevalence. Additionally, the presence of a stoma in CD patients following surgical interventions may further impact metabolic homeostasis and potentially increase the risk of NAFLD [22]. However, further research is required to investigate the specific contributions of treatment modalities and surgical interventions to NAFLD risk in CD and UC patients.

Environmental and lifestyle factors also differ across regions and could contribute to the varying prevalence rates. Dietary patterns, such as the consumption of high-calorie, high-fat diets, and excessive alcohol intake, are known risk factors for NAFLD. Variations in dietary habits and cultural practices across Europe, including the consumption of processed foods and sugary beverages, may contribute to a higher prevalence of NAFLD among CD and UC patients in this region [23]. Additionally, sedentary lifestyles and lack of physical activity, can further contribute to metabolic disturbances and increase the risk of NAFLD [24].

Genetic factors could also have a role in the observed regional disparities. Different populations have distinct genetic variations that may influence susceptibility to NAFLD. Variations in genes involved in lipid metabolism, insulin signaling, and inflammation pathways have been associated with the development and progression of NAFLD [25]. That's why a higher predisposition to NAFLD among CD and UC patients may be caused by the genetic makeup of the European population.

Healthcare infrastructure and access to healthcare services may also differ by region, leading to variations in disease detection and reporting [26]. Differences in diagnostic practices, screening protocols, and healthcare-seeking behavior among CD and UC patients can impact the identification and documentation of NAFLD cases. Variations in healthcare systems, such as the availability of specialized care and resources for managing liver disease, could also have an impact on the reported prevalence rates.

Another aspect to consider is the relatively lower overall prevalence of NAFLD in the Asian population compared to other regions. Studies have reported a lower prevalence of NAFLD in Asian countries [8,10], which could contribute to a narrower gap in NAFLD prevalence between CD and UC patients. The lower overall burden of NAFLD in the Asian population might be due to differences in dietary patterns, lifestyle factors, and genetic factors that influence the development of NAFLD.

The findings of this systematic review add significantly to the body of evidence associating CD and UC with NAFLD, offering light on the higher risk of NAFLD associated with CD versus UC. These findings highlight the importance of multidisciplinary interactions between gastroenterologists, hepatologists, and researchers in order to probe deeper into the complex interplay between these disorders. Also, identifying specific risk factors linked with the development of NAFLD in CD and UC populations, such as the function of gut microbiota, immunological dysregulation, and inflammatory processes, will provide vital insights into the underlying mechanisms and prospective treatment targets. More importantly, greater sample sizes and longer follow-up periods in longitudinal studies would allow for a more robust assessment of the temporal association between CD, UC, and NAFLD.

This systematic review's ramifications go beyond research, affecting both clinical practice and public health. Healthcare providers, including gastroenterologists and hepatologists, should be vigilant in assessing NAFLD risk factors in CD and UC patients, such as obesity, diabetes, dyslipidemia, and metabolic syndrome, and can use the results to inform targeted screening strategies, personalized monitoring approaches, and optimized treatment interventions for this population. Given the risk of severe consequences, such as liver fibrosis, cirrhosis, and hepatocellular carcinoma, early identification and therapy of NAFLD in CD and UC patients is critical [12]. Given the increased risk of NAFLD in CD patients, routine screening for NAFLD should be incorporated into CD management procedures, guaranteeing regular follow-ups and comprehensive care plans that address both the gastrointestinal and hepatic elements of these disorders. This knowledge can be used by public health initiatives to design targeted strategies for encouraging healthy lifestyle choices and lowering the burden of NAFLD in people with CD and UC. Collaboration between gastroenterology, hepatology, and primary care teams, as well as multidisciplinary approaches involving nutritionists and lifestyle counselors, may aid in optimizing patient management and lowering the risk of NAFLD.

Despite the useful information offered by this systematic review, certain limitations should be noted. First, the studies included were mostly observational, which has inherent limitations such as potential confounding factors and biases such as recall, information, selection, subjective, and so on. Second, the included studies varied in terms of study design, sample sizes, and diagnostic criteria, which could introduce heterogeneity and potential bias. Third, we could not account for the type of treatment that CD or UC patients received. Fourth, other possible confounders, such as concomitant CD or UC drugs (especially biologics), comorbidities, and disease flares, could not be taken into consideration. Additionally, the search was limited to English-language publications, potentially introducing language bias. Last, but not least, it's important to keep in mind that the systematic review only compared the prevalence of NAFLD in CD patients and UC patients and did not look at the causal link between CD, UC, and NAFLD, despite the fact that it offers insightful information about the prevalence of NAFLD in CD and UC patients across various regions. Future prospective studies and well-designed clinical trials are needed to better understand the temporal and causal associations between CD, UC, and NAFLD. Further research is necessary to clarify the correlations and uncover the underlying mechanisms between these conditions, employing large-scale investigations with standardized methodologies. Exploring environmental, genetic, and lifestyle factors that contribute to NAFLD prevalence in CD and UC populations across different regions should be a focus of future studies. 

Additionally, examining the impact of disease duration, severity, and treatment modalities on NAFLD development in CD and UC patients would provide a more comprehensive understanding. Comparative studies analyzing treatment outcomes, surgical techniques, and patient adherence to medical advice are needed to evaluate specific therapies and their regional variations. Overall, comprehending these variances and the interplay between CD, UC, and NAFLD is crucial for optimizing patient management and developing tailored strategies to reduce NAFLD's risk and burden in these populations across diverse geographical areas. Despite the limitations, we included a large number of studies with a diverse and robust number of patients.

## Conclusions

The greater prevalence rates of NAFLD among CD patients across different geographic regions indicate that CD has a stronger connection with NAFLD than UC. Regional differences are most likely influenced by genetic, lifestyle, and environmental variables. Variations in CD and UC disease features, inflammatory processes, and treatment methods may potentially have a role. The transmural inflammation in CD, as well as the potential involvement of the entire gastrointestinal tract, may raise the risk of metabolic diseases, including NAFLD. These findings emphasize the importance of doctors and healthcare workers being aware of the increased risk of NAFLD in CD patients and implementing appropriate monitoring and preventative strategies. To the best of our knowledge, this is the first systematic review to investigate whether Crohn’s disease is a more significant risk factor for nonalcoholic fatty liver disease compared to ulcerative colitis. Further research using standardized procedures, larger sample sizes, and prospective designs is necessary to understand the underlying mechanisms and develop personalized treatments for CD and UC patients, ultimately reducing the burden of NAFLD. This systematic review also serves as a foundation for future research and clinical decision-making, ultimately leading to better patient outcomes and overall care of these complicated illnesses.
